# Genotyping Reveals High Clonal Diversity and Widespread Genotypes of *Candida* Causing Candidemia at Distant Geographical Areas

**DOI:** 10.3389/fcimb.2020.00166

**Published:** 2020-05-05

**Authors:** Jesús Guinea, Maiken C. Arendrup, Rafael Cantón, Emilia Cantón, Julio García-Rodríguez, Ana Gómez, Elia Gómez G. de la Pedrosa, Rasmus K. Hare, Beatriz Orden, Maurizio Sanguinetti, Javier Pemán, Brunella Posteraro, Alba Ruiz-Gaitán, Gabriella Parisi, Daniel Archimedes Da Matta, Arnaldo L. Colombo, Carlos Sánchez-Carrillo, Elena Reigadas, Patricia Muñoz, Pilar Escribano

**Affiliations:** ^1^Clinical Microbiology and Infectious Diseases, Hospital General Universitario Gregorio Marañón, Madrid, Spain; ^2^Instituto de Investigación Sanitaria Gregorio Marañón, Universidad Complutense de Madrid, Madrid, Spain; ^3^CIBER de Enfermedades Respiratorias (CIBERES CB06/06/0058), Madrid, Spain; ^4^Unit of Mycology, Statens Serum Institut, Copenhagen, Denmark; ^5^Department of Clinical Microbiology, Rigshospitalet, Copenhagen, Denmark; ^6^Department of Clinical Medicine, University of Copenhagen, Copenhagen, Denmark; ^7^Servicio de Microbiología. Hospital Ramón y Cajal, Madrid and Instituto Ramón y Cajal de Investigación Sanitaria (IRYCIS), Madrid, Spain; ^8^Red Española de Investigación en Patología Infecciosa (REIPI), Instituto de Salud Carlos III, Madrid, Spain; ^9^Instituto de Investigación Sanitaria La Fe, Universidad de Valencia, Valencia, Spain; ^10^Department of Clinical Microbiology, Hospital La Paz, Madrid, Spain; ^11^Department of Clinical Microbiology, Hospital Puerta de Hierro-Majadahonda, Madrid, Spain; ^12^Dipartimento di Scienze di Laboratorio e Infettivologiche, Fondazione Policlinico Universitario A. Gemelli IRCCS, Rome, Italy; ^13^Department of Clinical Microbiology, Hospital Universitario y Politécnico La Fe, Valencia, Spain; ^14^Dipartimento di Scienze Gastroenterologiche, Endocrino-Metaboliche e Nefro-Urologiche, Fondazione Policlinico Universitario A. Gemelli IRCCS, Rome, Italy; ^15^Department of Clinical Microbiology, Azienda Ospedaliera San Camillo-Forlanini, Rome, Italy; ^16^Special Mycology Laboratory, Universida de Federalde São Paulo, São Paulo, Brazil; ^17^Medicine Department, Faculty of Medicine, Universidad Complutense de Madrid, Madrid, Spain

**Keywords:** *Candida*, genotyping, microsatellite, cluster, widespread

## Abstract

The objectives of this study were to gain further insight on *Candida* genotype distribution and percentage of clustered isolates between hospitals and to identify potential clusters involving different hospitals and cities. We aim to genotype *Candida* spp. isolates causing candidemia in patients admitted to 16 hospitals in Spain, Italy, Denmark, and Brazil. Eight hundred and eighty-four isolates (*Candida albicans, n* = 534; *C. parapsilosis, n* = 282; and *C. tropicalis, n* = 68) were genotyped using species-specific microsatellite markers. CDC3, EF3, HIS3, CAI, CAIII, and CAVI were used for *C. albicans*, Ctrm1, Ctrm10, Ctrm12, Ctrm21, Ctrm24, and Ctrm28 for *C. tropicalis*, and CP1, CP4a, CP6, and B for *C. parapsilosis*. Genotypes were classified as singletons (genotype only found once) or clusters (same genotype infecting two or more patients). Clusters were defined as intra-hospital (involving patients admitted to a single hospital), intra-ward (involving patients admitted to the same hospital ward) or widespread (involving patients admitted to different hospitals). The percentage of clusters and the proportion of patients involved in clusters among species, genotypic diversity and distribution of genetic diversity were assessed. Seven hundred and twenty-three genotypes were detected, 78 (11%) being clusters, most of which (57.7%; *n* = 45/78) were intra-hospital clusters including intra-ward ones (42.2%; *n* = 19/45). The proportion of clusters was not statistically different between species, but the percentage of patients in clusters varied among hospitals.

A number of genotypes (7.2%; 52/723) were widespread (found at different hospitals), comprising 66.7% (52/78) of clusters, and involved patients at hospitals in the same city (*n* = 21) or in different cities (*n* = 31). Only one *C. parapsilosis* cluster was a widespread genotype found in all four countries. Around 11% of *C. albicans* and *C. parapsilosis* isolates causing candidemia are clusters that may result from patient-to-patient transmission, widespread genotypes commonly found in unrelated patients, or insufficient microsatellite typing genetic discrimination.

## Introduction

Among invasive fungal infections, candidemia is the most common condition in hospitalized patients, representing an overall incidence rate of 3.88 cases per 100,000 hospital admissions (Koehler et al., 2019). With *Candida albicans* on the top of etiological agents of candidemia, infections with other species, including *Candida parapsilosis, Candida tropicalis*, and *Candida glabrata* are on the rise (Lamoth et al., 2018; Pappas et al., 2018). Prevalence of candidemia in high-risk patients such as critical care patients, oncology, and solid organ transplant recipients ranges from 2 to 11% (Ostrosky-Zeichner et al., 2007; Eggimann et al., 2011). Candidemia is associated with high mortality and high inpatient cost (Wan Ismail et al., 2019). Attributable mortality ranges from 11 up to 47% (Bilir et al., 2015).

Candidemia is a nosocomial infection that affects patients with diverse underlying conditions often caused by the use of intravascular catheters or translocation of endogenous isolates from the gut microbiota to the bloodstream (Puig-Asensio et al., 2014). Exogenous patient-to-patient transmission may represent an alternative route of infection. Genotyping may help clarify potential *Candida* outbreaks and transmission in hospitalized patients (Escribano et al., 2013, 2018) and clarify if certain genotypes occur in different patients—namely clusters—potentially suggesting a common isolate niche (Escribano et al., 2013; Hammarskjold et al., 2013).

We have previously shown different percentages of clustered isolates in Spanish and Italian hospitals, suggesting dissimilar infection control policies (Marcos-Zambrano et al., 2015). For example, campaigns to reduce the number of catheter-related infections led to a decrease in the number of clusters (Escribano et al., 2013, 2018). However, some clusters involved patients who were either admitted to the same hospital but without a clear epidemiological link (Escribano et al., 2013, 2018) or admitted to different hospitals that in occasions were located in different countries (Marcos-Zambrano et al., 2015). Such “epidemiologically unexplainable” clusters may indicate a limited discriminatory potential of the typing method or common widespread clones rather than active patient-to-patient transmission.

The aim of this study is to genotype *Candida* spp. isolates causing candidemia in subjects admitted to 16 hospitals in Spain, Italy, Denmark, and Brazil with high or very low likelihood of having been in contact. We provide insight into the genotypes and percentages of clustered isolates between these hospitals, and identify clusters involving different hospitals and cities.

## Materials and Methods

### Participating Hospitals and Clinical Isolates

Sixteen tertiary hospitals located in Spain (Madrid, *n* = 4; Valencia, *n* = 1), Italy (Rome, *n* = 2), Brazil (São Paulo, *n* = 1), and Denmark (three clinical microbiological laboratories that together served 8 university hospitals in Copenhagen) participated in this multicentre study. Isolates were collected from consecutive episodes of candidemia (one isolate per patient; *n* = 884) caused by *C. albicans* (*n* = 534), *C. parapsilosis* (*n* = 282), or *C. tropicalis* (*n* = 68) diagnosed in patients admitted to the above-mentioned hospitals between January 2014 and December 2015. Isolates were identified by MALDI-TOF mass spectrometry or molecular methods (White et al., 1990; Marklein et al., 2009; De Carolis et al., 2014; Normand et al., 2019). The number of admissions, incidence and number of episodes per species during the study period are shown in Table 1. All participating hospitals have active taskforces, including microbiologists, infectious disease specialists and nurses, to monitor and control infections, and have implemented antimicrobial stewardship programs or, so called, zero bacteraemia programs. Details of such campaigns at each hospital were not available.

**Table 1:** Epidemiology of candidemia for each participating hospital during the study period.

**Hospital**	**2014-2015 period**			**No. of incident episodes/isolates studied**		
	**No. of admissions**	**Cases of candidemia**	Incidence of candidemia^*^	***C. albicans***	***C. parapsilosis***	***C. tropicalis***
**Brazil**						
São Paulo	47,176	153	3.06	78/61	31/14	23/19
Denmark^**^						
Rigshospitalet	184,216	122	0.66	58/55	9/7	6/5
Herlev	510,335	108	0.21	55/51	5/3	6/6
Hvidovre	322,090	94	0.29	31/26	3/3	6/5
**Spain**						
Gregorio Marañón	96,890	116	1.17	51/51	32/32	4/4
La Fe	77,833	148	1.19	46/43	64/49	4/4
La Paz	93,044	100	1.08	38/7	36/29	3/2
Puerta de Hierro	51,573	72	1.39	30/26	26/24	2/1
Ramón y Cajal	63,432	85	1.30	36/30	31/29	3/2
**Italy**						
Fondazione Policlinico A. Gemelli	155,851	312	1.99	156/143	74/73	13/13
San Camillo	129,470	340	1.85	45/41	21/19	8/7
**Overall**				624/534	332/282	78/68

* *Mean incidence (cases of candidemia per 1,000 hospital admissions) during 2014–2015*.

** *Hospitals located at Copenhagen: Bispebjerg, Frederiksberg, Gentofte, Glostrup, Herlev, Hillerød, Hvidovre, Rigshospitalet*.

### Genotyping

Isolates were genotyped by species-specific microsatellite markers. Six markers were used for *C. albicans*: CDC3, EF3, HIS3 (Botterel et al., 2001; Sabino et al., 2010), CAI, CAIII, and CAVI (Sampaio et al., 2005). Markers used for *C. tropicalis* were Ctrm1, Ctrm10, Ctrm12, Ctrm21, Ctrm24, and Ctrm28 (Wu et al., 2014), and CP1, CP4a, CP6, and B were used for *C. parapsilosis* (Sampaio et al., 2003; Vaz et al., 2011).

Capillary electrophoresis using the ABI 3130xl analyser (Applied Biosystems-Life Technologies Corporation, Carlsbad, California, USA) and the GeneScan ROX 500 bp marker (Applied Biosystems-Life Technologies Corporation, Carlsbad, California, USA) was performed on the PCR products. Electropherograms were analyzed with the GeneMapper® v.4.0 software (Applied Biosystems-Life Technologies Corporation, California). A control strain from each species was used in each run to ensure size accuracy and avoid run-to-run variations. The number of base pairs determined the size of alleles in each locus.

### Genotype and Cluster Analysis

Allele results were converted to binary data by scoring the presence or absence of each allele. Data were treated as categorical, and the genetic relationship between genotypes was examined by constructing a minimum spanning tree (BioNumerics version 6.6, Applied Maths, Sint-Martens-Latem, Belgium). Isolates were considered to have identical genotypes when they showed the same alleles in all loci. Different genotypes were codified as follows: CA-X (*C. albicans*), CP-X (*C. parapsilosis*), and CT-X (*C. tropicalis*), X representing the internal code of the genotype in our collection. Definitions of singleton genotypes and clusters are summarized in Table 2.

**Table 2:** Definitions of singleton genotypes, clusters and clonal complexes of the genotypes used in this study.

**Term**	**Definition**	**Possible interpretation**
Singleton	Genotype found only once	
Cluster	Same genotype infecting ≥2 patients (with or without an epidemiological link between the involved patients)	Potentially indicating transmission, widespread clones or genetic differences not detected by the genotyping method
Intra-hospital cluster	Same genotype involving ≥2 patients admitted to the same hospital within a period of 12 months	Genotype endemic in the hospital that may infect patients not necessarily during their stay in their admission wards
Intra-ward cluster	Intra-hospital cluster involving ≥2 patients admitted to the same hospital ward within a period of 12 months	Genotype likely indicating the occurrence of patient-to-patient transmission in a given hospital ward
Widespread cluster	Same genotype involving ≥2 patients admitted to different hospitals	Genotype that usually infects unrelated patients and that barely indicates patient-to-patient transmission
Intra-hospital and widespread cluster	Intra-hospital genotypes found in at least one more patient at another hospital	Genotype widely distributed usually infecting unrelated patients; barely indicates patient-to-patient transmission
Clonal complex	Group of genotypes showing differences in a single locus	Group of clusters genetically related among them, frequently found and that may have evolved from a common ancestor

We compared the percentage of clusters and the proportion of patients presenting clusters among species and hospitals using a standard binomial method (95% confidence intervals) (Epidat 3.1 software, Servicio de Información sobre Saúde Pública de la Dirección Xeral de Saúde Pública de la Consellería de Sanidade, Xunta de Galicia, Spain).

### Genotypic Diversity Analysis

We assessed the following diversity parameters: (a) number of alleles per locus; (b) observed (direct count) heterozygosity (Ho), calculated as the number of heterozygous genotypes over the total number of genotypes analyzed for each locus; (c) expected heterozygosity (He; calculated as He = 1–Σpi^2^, where pi represents the frequency of the i^th^ allele) (Nei, 1973); Wright's fixation index [*F* = 1– (Ho/He)] shows the relationship between Ho and He and detects heterozygote excess or deficiency (Lenardon and Nantel, 2012); and (d) the probability of identity among unrelated individuals [PI= 1–Σpi^4^ + ΣΣ(2pipj)^2^], where *pi* and *pj* represent the frequencies of the i^th^ and j^th^ alleles, respectively and measures the likelihood that two randomly drawn diploid genotypes will be identical, assuming the observed allele frequencies and random assortment (Paetkau et al., 1995). Significant deviations from the Hardy-Weinberg equilibrium (HWE) at individual loci were tested using the Markov chain method to determine if a population was clonal. For the analyses the IDENTITY 1.0 (Wagner et al., 1999) and ARLEQUIN version 3.01 (Excoffier et al., 2005) programs were used.

### Distribution Analysis of Genetic Diversity

Molecular variance analysis AMOVA) was performed to determine the distribution of genetic diversity based on the number of alleles with 10,000 permutations (ARLEQUIN version 3.01) (Excoffier et al., 2005). AMOVA (term and model inspired by ANOVA but adapted to molecular data) is a statistical model that allows estimating population differentiation directly from molecular data. It tests hypotheses on such differentiations for the molecular algorithm in a single species, usually biological. Different hierarchical levels—populations—were established among countries, between singleton genotypes and clusters (overall and clusters grouped by countries). *F* statistics for each hierarchical level were computed. Pairwise *FST* values obtained from AMOVA were used to measure the genetic differentiation between populations and its significance (*P* < 0.05) was assessed using a non-parametric permutation approach (Excoffier et al., 2005). Pairwise *FST* values between countries were represented by Principal Coordinate Analysis (PCoA) with the GENEALEX software (Peakall and Smouse, 2012). PCoA allows exploring and visualizing data similarities or dissimilarities.

## Results

We detected 723 genotypes distributed as follows: *C. albicans* (*n* = 453), *C. parapsilosis* (*n* = 206), and *C. tropicalis* (*n* = 64); 645 (89.2%) were singletons and 78 (10.8%) were clusters (Table S1 and Table 3). The proportion of clusters was not statistically different between species [*C. albicans*, 10.6% (48/453), *C. parapsilosis*, and 12.6% (26/206), and *C. tropicalis*, 6.2% (4/64)]. The 78 clusters were intra-hospital (*n* = 26), widespread (*n* = 33) or intra-hospital/widespread (*n* = 19).

**Table 3:** Number of singleton genotypes and intra-hospital/intra-ward clusters found in each participating hospital.

**Hospital**	***C. albicans*** **(N**^****°****^**)**				***C. parapsilosis*** **(N**^****°****^**)**				***C. tropicalis*** **(N**^****°****^**)**			
	**Genotypes**	**Singleton**	**Clusters**	**Intra-ward/intra-hospital clusters**	**Genotypes**	**Singleton**	**Clusters**	**Intra-ward/intra-hospital clusters**	**Genotypes**	**Singleton**	**Clusters**	**Intra-ward/intra-hospital clusters**
**Brazil**												
São Paulo	59	57	2	0/2	11	10	1	0/1	18	17	1	1/1
**Denmark**												
Rigshospitalet	50	47	3	0/3	7	7	0	0/0	5	5	0	0/0
Herlev	24	22	2	0/0	3	3	0	0/0	5	4	1	0/0
Hvidovre	51	51	0	0/0	3	3	0	0/0	5	5	0	0/0
**Spain**												
Gregorio Marañón	49	47	2	2/2	30	28	2	0/2	4	4	0	0/0
La Fe	39	35	4	1/4	36	31	5	1/5	4	4	0	0/0
La Paz	7	7	0	0/0	25	22	3	1/3	2	2	0	0/0
Puerta de Hierro	26	26	0	0/0	22	21	1	1/1	1	1	0	0/0
Ramón y Cajal	29	28	1	0/1	21	17	4	2/4	2	2	0	0/0
**Italy**												
Fondazione policlinico A. Gemelli	122	109	13	4/13	59	53	6	5/6	12	11	1	1/1
San Camillo	40	39	1	0/1	19	19	0	0/0	7	7	0	0/0
**Overall**	453	405	48	7/26	206	180	26	10/22	64	60	4	2/2

**Figure 1:**
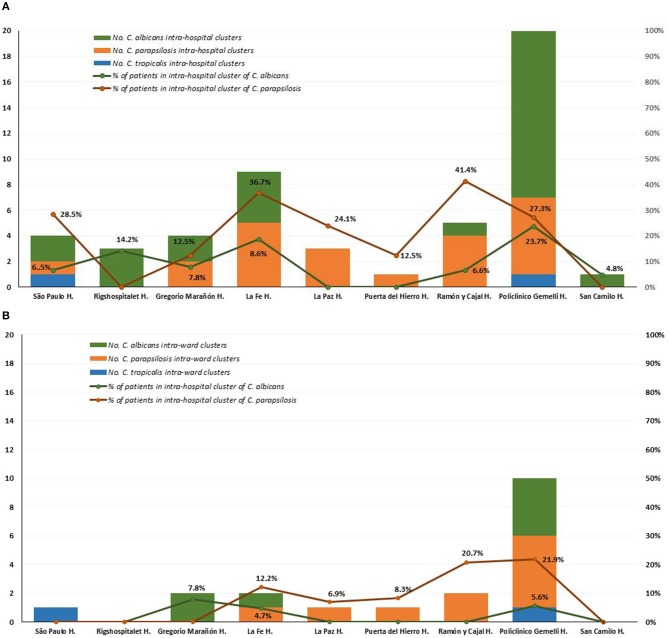
Distribution of intra-hospital clusters **(A)**, intra-ward clusters **(B)**, and percentage of patients involved in clusters among participating hospitals.

Intra-hospital clusters (including intra-hospital/widespread clusters) comprised around 57% (*n* = 45/78) of the clusters (*C. albicans, n* = 26/48; *C. parapsilosis, n* = 22/26; and *C. tropicalis, n* = 2/4) and were found mostly in Fondazione Policlinico A. Gemelli (*n* = 20), La Fe (*n* = 9), and Ramón y Cajal (*n* = 5) hospitals, but were scarcely found in Copenhagen hospitals (Tables 3, 4, and Figure 1). One hundred and thirty-four patients presented intra-hospital clusters (*C. albicans, n* = 62; *C. parapsilosis, n* = 68; *C. tropicalis, n* = 4). The proportion of patients infected by *C. parapsilosis* clusters in most hospitals was higher compared with *C. albicans* (Figure 1A). The 45 intra-hospital clusters were reduced to 19 intra-ward clusters (*C. albicans, n* = 7; *C. parapsilosis, n* = 10; *C. tropicalis, n* = 2) that involved 50 patients (*C. albicans, n* = 14; *C. parapsilosis, n* = 32; *C. tropicalis, n* = 4), half of which were detected at the medical wards of Fondazione Policlinico A. Gemelli hospital (Table 5 and Figure 1B).

**Table 4:** Intra-hospital clusters indicating the species, number of patients involved in each cluster and affected hospitals.

**Species**	**Cluster code**	**Hospital**	**Patients involved**	**Species**	**Cluster code**	**Hospital**	**Patients involved**
***C. albicans***	CA-789	São Paulo	2	***C. parapsilosis***	CP-039^*^	H. Fondazione A. Gemelli	2
	CA-830		2		CP-355	São Paulo	4
	**CA-039**	Gregorio Marañón	2		CP-056^*^	Gregorio Marañón	2
	**CA-406**		2		CP-023^*^	Gregorio Marañón	2
	CA-305	La Fe	2			La Fe	2
	**CA-432**		2			Fondazione A. Gemelli	5
	CA-502		2			Ramón y Cajal	2
	CA-515^*^		2		CP-031^*^	La Fe	8
	CA-035^*^	H. Fondazione A. Gemelli	2		CP-063		3
	CA-048^*^		4		CP-161^*^		2
	CA-051^*^		3		CP-117^*^	La Fe	3
	CA-059^*^		2			La Paz	3
	**CA-071**		3		**CP-065**	La Paz	2
	CA-107		2		CP-094^*^		2
	CA-364^*^		3		CP-196^*^	Puerta de Hierro	3
	CA-632		3			Ramón y Cajal	3
	CA-633		3		CP-199^*^	H. Fondazione A. Gemelli	2
	CA-670		3		**CP-271**		4
	CA-701		2		**CP-272**		3
	CA-736^*^		2		**CP-282**		4
	CA-762		2		**CP-240**	Ramón y Cajal	3
	CA-565^*^	Rigshospitalet	4		**CP-242**		4
	CA-571		2	***C. tropicalis***	**CT-074**	H. Fondazione A. Gemelli	2
	CA-597^*^		2		**CT-118**	São Paulo	2
	CA-331^*^	Ramón y Cajal	2				
	CA-741	San Camilo	2				

* *Intra-hospital and widespread clusters*.

*Cluster code in bold indicates intra-ward cluster and the hospital at which they were found; genotypes CP-023 and CP-065 are widespread clusters and also intra-ward clusters in Fondazione A. Policlinico Gemelli and Puerta de Hierro hospitals, respectively*.

**Table 5:** Intra-ward clusters indicating the species, number of patients involved in each cluster, and affected hospitals and wards.

**Species**	**Cluster code**	**Patients involved in intra-ward clusters**	**Hospital**	**Admission ward**	**Date of blood culture collection**
***C. albicans***	CA-039	2	Gregorio Marañón	Post-surgical ICU	Oct 2014 Dec 2014
	CA-406	2	Gregorio Marañón	Neonatology	Sep 2015 Sep 2015
	CA-432	2	La Fe	Hematology	Sep 2014 Oct 2014
	CA-048	2	Fundazione Policlinico A. Gemelli	Medical ward	Sep 2015 Nov 2015
	CA-059	2	Fundazione Policlinico A. Gemelli	Medical ward	Apr 2014 Jun 2014
	CA-071	2	Fundazione Policlinico A. Gemelli	Medical ward	Feb 2014 Feb 2014
	CA-364	2	Fundazione Policlinico A. Gemelli	Medical ward	May 2014 May 2014
***C. parapsilosis***	CP-023	3	Fundazione Policlinico A. Gemelli	Medical ward	Jan 2015 Jul 2015 Aug 2015
	CP-031	3 3	La Fe	Post-surgical ICU	Jul 2014 Jul 2014 Feb 2015 Jul 2015 Jul 2015 Nov 2015
	CP-065	2	La Paz	Post-surgical ICU	Jul 2015 Dec 2015
	CP-196	2	Puerta de Hierro	ICU	Nov 2015 Nov 2015
	CP-199	2	Fundazione Policlinico A. Gemelli	Medical ward	Dec 2014 Dec 2014
	CP-271	4	Fundazione Policlinico A. Gemelli	Pneumology	May 2015 Jul 2015 Sep 2015 Dec 2015
	CP-272	3	Fundazione Policlinico A. Gemelli	Pneumology	Sep 2014 Nov 2014 Apr 2015
	CP-282	4	Fundazione Policlinico A. Gemelli	Neonatal ICU	Nov 2014 Dec 2014 Jan 2015 Oct 2015
	CP-240	2	Ramón y Cajal	Hematology	Oct 2015 Nov 2015
	CP-242	4	Ramón y Cajal	Surgical ward	Aug 2014 Sep 2014 Oct 2014 Nov 2014
***C. tropicalis***	CT-074	2	Fundazione Policlinico A. Gemelli	Medical ward	Jun 2015 Jun 2015
	CT-118	2	São Paulo	ICU	Aug 2014 Aug 2014

**Figure 2:**
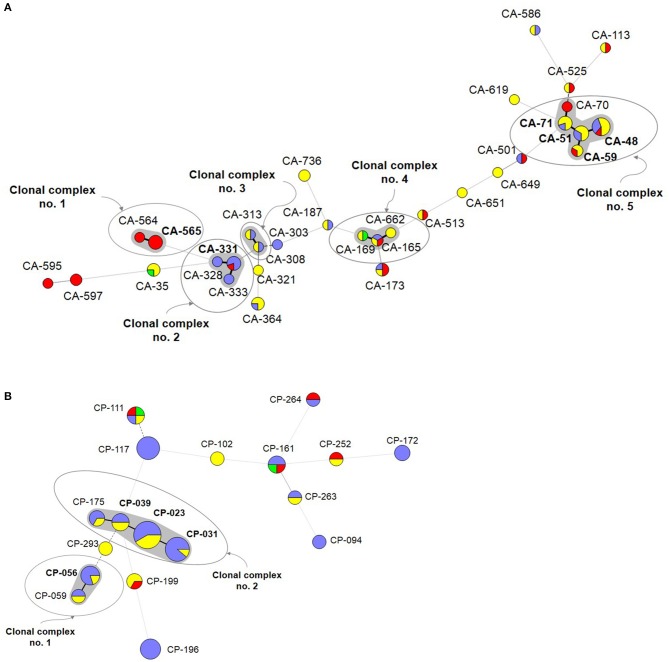
Minimum spanning tree showing the *C. albicans* **(A)** and *C. parapsilosis* **(B)** widespread genotypes. Circles represent different genotypes and circle size the number of isolates belonging to the same genotype. Colors indicate the country from which the isolate was obtained. Connecting lines between the circles show profile similarities; solid bold lines indicate differences in only 1 marker (clonal complexes are shadowed in gray), the solid line indicates differences in 2 markers, long dashes in dashed line indicate differences in 3 markers, and short dashes indicate differences in 4 or more markers. Genotypes in bold indicate widespread genotypes and intra-hospital clusters. Denmark (red), Spain (purple), Italy (yellow), and Brazil (green).

A number of genotypes (7.2%; 52/723) were widespread (*C. albicans, n* = 32/453; *C. parapsilosis, n* = 18/206; *C. tropicalis, n* = 2/64) and were occasionally found as intra-hospital clusters at some hospitals (Table 4). Overall, widespread genotypes comprised 52 of the 78 detected clusters and involved patients at hospitals in the same (*n* = 21) or in different cities (*n* = 31) (Figure 2); *C. tropicalis* widespread genotypes (*n* = 2) were found exclusively in two hospitals in Copenhagen. A number of widespread *C. albicans* clusters involved 2–9 patients each and five clonal complexes were found. Interestingly, at least one genotype fulfilling the definition of widespread and intra-hospital cluster was detected in four of the five clonal complexes (Figure 2A). Clonal complex number 5 included the highest number of clusters (*n* = 5) and patients (*n* = 25). It is worth noting that four out of the five clusters were widespread and intra-hospital clusters and many of them were found in Fondazione Policlinico A. Gemelli hospital. As for *C. parapsilosis* widespread genotypes, we found some clusters affecting several patients each (2–12 patients), whereas the number of clonal complexes was lower than those found for *C. albicans*. Again, at least one genotype fulfilling the definition of widespread and intra-hospital cluster was detected in each clonal complex (Figure 2B). Clonal complex number 2 was particularly significant due to the high number of genotypes (*n* = 4) and patients (*n* = 28) involved. Three out of the four clusters were widespread and intra-hospital clusters in different hospitals (Table 4). Only one *C. parapsilosis* cluster was a widespread genotype found in the four countries.

### Genetic Diversity and Analysis of Molecular Variance

Diversity parameters are shown in Table S2. The used microsatellite marker appeared to be highly discriminatory since the probability of identity for *C. albicans, C. parapsilosis* and *C. tropicalis* was very low (7.7 × 10^−10^, 1.2 × 10^−6^, and 4 × 10^−8^, respectively). Probability of identity values close to zero indicates high discriminative power of the used marker. In this work, total diversity of *C. albicans* isolates was elevated as shown by the high number of alleles found (*N* = 195; mean of 32.5 alleles/marker), the low frequency of null alleles (Na = 0.08), and the observed and expected heterozygosity (Ho = 0.67 and He = 0.84, respectively). Contrarily, the diversity of *C. parapsilosis* and *C. tropicalis* isolates was lower.

Wright's index (*F*) indicates a deficiency (positive values) or excess (negative values) heterozygosity. Heterozygote deficiency was observed in the three species (*C. albicans* [*F* = 0.19], *C. parapsilosis* [*F* = 0.43], and *C. tropicalis* [*F* = 0.38]). Allele frequencies of all loci differed significantly (*P* < 0.05) from what was expected in a population in Hardy–Weinberg equilibrium for the three species. This suggests clonal expansion due to migrations between countries/hospitals, although we should not exclude other factors including genetic drift, natural selection, sexual selection, mutation, gene flow, meiotic drive, genetic hitchhiking, population bottleneck, founder effect and inbreeding. Diversity parameters were assessed by country with no significant differences (Table S2).

The AMOVA analysis per species showed that most estimated variations were found within the strains of a given population; however, pooling isolates by geographical regions showed that a small but significant proportion of variations may be attributed to differences among countries (3% *C. albicans*, 2.22% *C. parapsilosis*, and 5.63% *C. tropicalis, P* < 0.001) (Table 5). Likewise, this analysis revealed differences between singleton genotypes and clusters (1.5% *C. albicans*, 1% *C. parapsilosis*, and 5.96% *C. tropicalis, P* < 0.05) (Table 6).

**Table 6:** Analysis of molecular variance (AMOVA)^*^ partitioning genetic diversity within and among countries, and within and between singletons and clusters.

**Source of variation**	***C. albicans***		***C. parapsilosis***		***C. tropicalis***	
	**% of variation**	FST^*^	**% of variation**	***FST***	**% of variation**	***FST***
Among countries	3.01	**0.03**	2.22	**0.02**	5.63	**0.05**
Within countries	96.99		97.78		94.37	
Between singletons/clusters	1.50	**0.01**	1.04	**0.01**	5.96	**0.05**
Within singletons/clusters	98.50		98.96		94.04	

* *AMOVA values measure the genetic differentiation between populations. Numbers shown in bold indicate statistically significant differences (P < 0.05)*.

**Figure 3:**
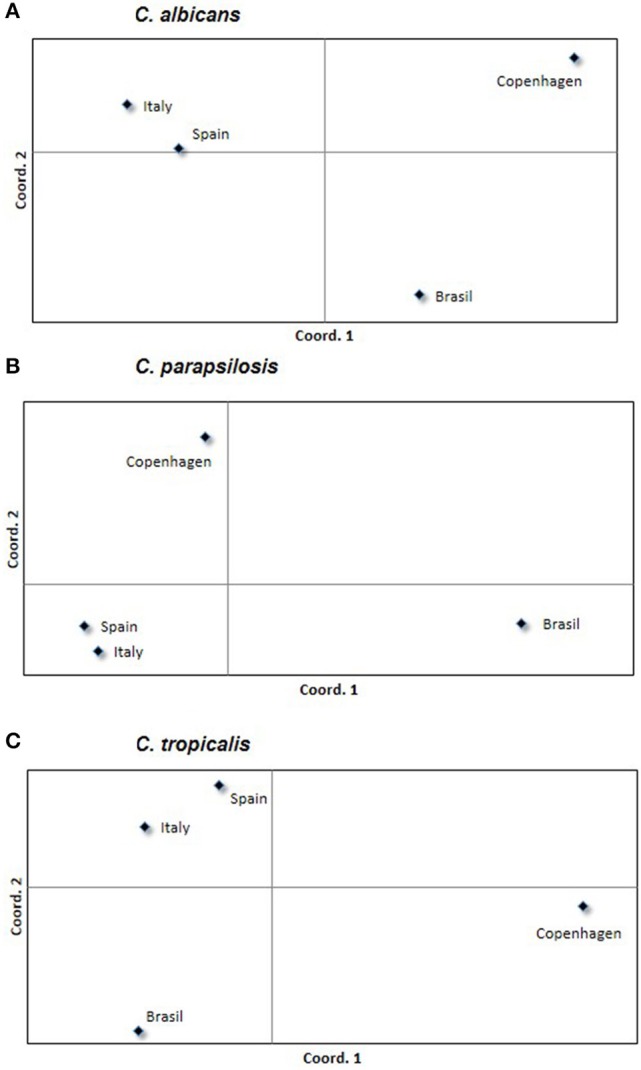
Principal coordinates analysis showing the genetic differentiation of *C. albicans* **(A)**, *C. parapsilosis* **(B)**, and *C. tropicalis* **(C)** among isolates grouped by country. Symbols represent the variability found within each country; symbols in the same square are more likely to be related among them rather than to those located in other squares.

*FST* measures the genetic differentiation between populations with lower values indicating scarce differentiation. *C. tropicalis* showed the highest differentiation among countries (*FST* = 0.05, *P* < 0.001) and between singletons and clusters (*FST* = 0.05, *P* < 0.05). Principal coordinate representation of *FST* values for the three species shows low but significant differentiation between countries, with isolates from Spain and Italy being more closely related (Figure 3).

## Discussion

The genotyping analysis performed in this study reveals widespread *Candida* genotypes causing candidemia at distant geographic areas.

Previous studies suggest that *Candida* genotyping is useful to study hospital infection outbreaks under the umbrella of clinical suspicion (Diekema et al., 1997; Kuhn et al., 2004; Lasheras et al., 2007). Conversely, blindly genotyping of consecutive candidemia isolates unveiled lurking genotypes actively causing infections at some wards (Escribano et al., 2013) where implementation of campaigns to prevent catheter-related infections had a positive impact (Escribano et al., 2018). Moreover, differences in the number of clusters among hospitals may indicate dissimilar infection control policies with potential room for improvement (Marcos-Zambrano et al., 2015). Results from our previous abovementioned studies led us to conclude that the higher the incidence of candidemia in a given period of time, the higher the percentage of clusters. Additionally, some clusters involve epidemiologically linked patients (same ward within 12 months) suggesting either nosocomial transmission between patients or outbreaks, whereas other clusters involve patients who are apparently epidemiologically unrelated. The presence of these unexplainable clusters led us to coin the term “widespread genotypes,” making reference to patients admitted to different hospitals. Here, we enlarge the number of isolates and participating hospitals located at various geographic regions that would resemble differences in hospital management and variable candidemia incidence.

The prevalence of all three *Candida* species is consistent with previously reported candidemia epidemiology (Colombo et al., 2017). Even though most patients in this study were infected by singleton genotypes (89%), clusters were detected in around 11% of them. Notably, the proportion of patients with clusters and incidence of candidemia differed between hospitals, with the lowest incidence found in Denmark and the highest in Brazil, which may indicate distinct campaigns to control hospital infections, adherence to infection control guidelines, type of population at risk of acquiring candidemia in each hospital, and/or differences in patient length of stay at the health care center. The result of candidemia incidence does not necessarily match with the number/proportion of clusters. *C. tropicalis* and *C. albicans* are, respectively, the species with the lowest and highest number of clusters, and *C. parapsilosis* (*n* = 17) presenting an intermediate number. When intra-ward clusters are analyzed, the number of *C. albicans* clusters outnumbers those of *C. parapsilosis* (*n* = 7 vs. *n* = 10). This indicates that *C. parapsilosis* clusters resist a more stringent definition of cluster and may show patient-to-patient transmission. Candidemia-related outbreaks by *C. parapsilosis* are relatively common (Diab-Elschahawi et al., 2012; Singh et al., 2019; Toth et al., 2019).

The genetic definition of cluster may be misleading when trying to disentangle hospital patient-to-patient transmission, particularly in apparently unrelated patients. When epidemiological information is considered, clusters may be tagged differently. The most restrictive definition of intra-ward cluster requires that the affected patients be admitted to the same hospital ward thereby increasing the likelihood of patient-to-patient transmission. Contrarily, intra-hospital clusters may indicate the presence of an endemic genotype in the hospital that infects patients admitted to different wards. An intra-hospital cluster not meeting the definition of intra-ward cluster is an enigma and may result from a lack of microsatellite discriminatory power. However, there are other possible explanations. First, given the retrospective nature of most studies, patients may have acquired the infection not in the niche of the genotype, as shown by *Clostridioides difficile* (Tarrant et al., 2018). Second, some genotypes may be more frequent and better adapted than others, persisting in hospital facilities for a longer time. Finally, we speculate that the higher the number of patients colonized by certain genotypes, the higher the chances to find the genotypes causing candidemia. Furthermore, the presence of widespread genotypes is even more difficult to interpret and we cannot rule out that certain clones may have spread worldwide. Some genotypes can be found in different countries, as reported with *Candida glabrata* (Al-Yasiri et al., 2016; Adams et al., 2018) or more recently on *C. auris* (de Groot et al., 2020).

*C. auris* was first detected in 2009 and since then different local clades have been identified. These clades are thought to be diverging locally over time. Coexistence of several geographic clades have identified at hospitals admitting patients from separate endemic areas of *C. auris* infections (Borman et al., 2017; de Groot et al., 2020). With this scenario, similar clones infecting different patients may not be an indication of patient-to-patient transmission but a previous colonization by the specific clade even before the admission of the patient to the hospital. Migration from endemic areas may facilitate clone dissemination (Al-Yasiri et al., 2016; Carrete et al., 2018; de Groot et al., 2020). As *C. albicans* and *C. parapsilosis* have had more time to evolve than *C. auris*, the number of genotypes found for these two species is higher than for *C. auris*. Widespread genotypes may resemble what is seen for the different *C. auris* linages. That is, widespread genotypes resemble clonal genotypes with longer time to spread worldwide by colonizing many individuals (de Groot et al., 2020), demonstrated the presence of identical *C. auris* genotypes in different countries.

The lack of geographic-specific genotypes shown by the AMOVA analysis points to a similar genetic background and a common clonal ancestor with an effective spread pattern. *C. albicans* and *C. parapsilosis* populations are not in Hardy-Weinberg equilibrium, suggesting that the genetic background in these species is mostly clonal or followed by selection pressure by interaction with the host or the environment (Sabino et al., 2010). Other markers, such as multilocus sequence typing, have previously suggested a clonal origin of *C. albicans* isolates (Tavanti et al., 2005).

There is no neat genotype geographic-specific distribution. However, genotypes from Italy and Spain are more similar among them in comparison to the others. This observation, which in turn may suggest a more frequent dissemination of genotypes between two neighboring countries, may be a consequence of the large number of isolates from the these two countries.

Our study has some limitations: First, we cannot rule out that some clusters may be a consequence of insufficient microsatellite typing discriminatory power—although diversity parameters do not support this—due to events of genomic recombination or partial chromosomal aneuploidy (Hirakawa et al., 2015). Whole genome sequencing of isolates of the largest clonal complexes are justified. Second, we missed some isolates causing candidemia during the study period, which prevented us from confirming all potential clusters. Third, we had an asymmetric collection of isolates as some countries contributed with more isolates than others. Fourth, we did not genotype environmental isolates that may be prevalent in the hospitals or assessed their frequency in those wards of hospitalization before the study period. Fifth, culture-negative cases of invasive candidiasis may lead to underestimate the number of clusters. Finally, clinical data was not collected, preventing us to decipher if clusters could be associated with specific clinical conditions or if they represented patient-to-patient transmission.

In conclusion, we show that around 11% of *C. albicans* and *C. parapsilosis* isolates causing candidemia are clusters originated from either patient-to-patient transmissions, widespread genotypes commonly found in unrelated patients, or lack of microsatellite genetic discrimination. Further studies using whole genome sequencing will help decipher the usefulness of microsatellite markers to conduct epidemiological studies and redefine the definition of cluster.

## Data Availability Statement

All relevant data is contained within the article. The original contributions presented in the study are included in the article/Supplementary Files, further inquiries can be directed to the corresponding authors.

## Ethics Statement

This study was approved by the Ethics Committee of Hospital Gregorio Marañón (CEIC-A1; study no. 108/16).

## Author's Note

This study was partially presented at the 27th European Congress of Clinical Microbiology and Infectious Diseases in Vienna (P1437 and P0947), Austria, April 2017 and at the 28th European Congress of Clinical Microbiology and Infectious Diseases in Madrid (EP863), Spain, April 2018.

## Author Contributions

PE and JG designed the study. AG contributed to the development and methodology. MA, RC, EC, JG, AG, EP, RH, BO, MS, JP, BP, AR-G, GP, DD, AC, CS-C, ER, PM, JG-R, and PE collected the data. JG and PE carried out the data analysis and investigation. MA, RC, PE, and JG wrote all sections of the manuscript. MA, RC, EC, JG, AG, EP, RH, BO, MS, JP, BP, AR-G, GP, DD, AC, CS-C, ER, PM, JG, and PE revised the manuscript.

## Study Group

The following participants are part of the project's study group: Jesus Guinea, Ana Gómez, Elena Reigadas, Patricia Muñoz, Carlos Sánchez-Carrillo, and Pilar Escribano (Hospital Gregorio Marañón, Madrid, Spain). Maiken C. Arendrup, Rasmus Hare (Statens Serum Institut, Copenhagen, Denmark). Rafael Cantón, Elia Gómez (Hospital Ramón y Cajal, Madrid, Spain). Emilia Cantón, Javier Pemán and Alba Cecilia Ruiz-Gaitán (Hospital La Fe, Valencia, Spain). Maurizio Sanguinetti, Brunella Posteraro and Antonietta Vella (Fondazione Policlinico Universitario A; Gemelli IRCCS, Rome, Italy). Gabriella Parisi (Azienda Ospedaliera San Camillo-Forlanini, Rome). Julio García-Rodríguez (Hospital La Paz, Madrid, Spain). Beatriz Orden (Hospital Puerta de Hierro, Madrid, Spain). Arnaldo Lopes Colombo, Daniel Archimedes Da Matta (Universidade Federal de São Paulo, São Paulo, Brazil).

## Conflict of Interest

JG has received funds for participating at educational activities organized on behalf of Astellas, Gilead, MSD, Scynexis, and Biotoscana-United Medical; he has also received research funds from FIS, Gilead, Scynexis, and Cidara, outside the submitted work. AC received educational grants from Biotoscana-United Medical, MSD, Pfizer and research grant from Astellas and Pfizer. MA reports personal fees from Astellas, Basilea, Gilead, MSD, Pfizer, T2Biosystems, and Novartis, other from Astellas, Basilea, Gilead, T2Biosystems, F2G, Novabiotics and Amplyx outside the submitted work. The remaining authors declare that the research was conducted in the absence of any commercial or financial relationships that could be construed as a potential conflict of interest. The reviewer JM declared past co-authorship with one of the authors AC to the handling editor.

## Supplementary Material

Table S1 Genotype code, type of genotype, and microsatellite allelic composition in base pairs.

Table S2 Diversity parameters calculated for each country as mean values. ^*a*^Observed and expected heterozygosity ranged from 0 (no heterozygosity) to 1(highest heterozygosity). ^*b*^Wright's index indicates a deficiency of heterozygosity (positive values) or excess heterozygosity (negative values). ^*c*^The probability of identity indicates the likelihood of finding two identical genotypes after randomly selecting two isolates.
