# Supplementary material for: Genotyping Reveals High Clonal Diversity and Widespread Genotypes of Candida Causing Candidemia at Distant Geographical Areas
Source: Front Cell Infect Microbiol. 2020 May 5;10:166. doi: 10.3389/fcimb.2020.00166 (PMC7214738; doi:10.3389/fcimb.2020.00166)
Supplement: Table S2 — Diversity parameters calculated for each country as mean values. aObserved and expected heterozygosity ranged from 0 (no heterozygosity) to 1(highest heterozygosity). bWright's index indicates a deficiency of heterozygosity (positive values) or excess heterozygosity (negative values). cThe probability of identity indicates the likelihood of finding two identical genotypes after randomly selecting two isolates. [file Table_2.DOCX]

| **Species** | **Country** | **Mean number of alleles** | **Null alleles** | **Observed heterozygosity^a^** | **Expected heterozygosity^a^** | **Wright's index^b^** | **Probability of identity^c^** |
| --- | --- | --- | --- | --- | --- | --- | --- |
| ***C. albicans*** | Brazil | 19.16 | 0.04 | 0.76 | 0.84 | 0.10 | 1.1x10^-9^ |
|  | Denmark | 23.67 | 0.10 | 0.67 | 0.85 | 0.22 | 3.1x10^-10^ |
|  | Italy | 25.50 | 0.08 | 0.65 | 0.81 | 0.17 | 5.4x10^-9^ |
|  | Spain | 24.50 | 0.06 | 0.69 | 0.81 | 0.14 | 7.1x10^-9^ |
|  | **Overall** | **32.50** | **0.08** | **0.68** | **0.84** | **0.19** | **7.7x10^-10^** |
| ***C. parapsilosis*** | Brazil | 9.00 | 0.14 | 0.53 | 0.79 | 0.31 | 1.5x10^-5^ |
|  | Denmark | 10.25 | 0.19 | 0.48 | 0.85 | 0.42 | 1.1x10^-6^ |
|  | Italy | 21.50 | 0.17 | 0.48 | 0.78 | 0.39 | 5.8x10^-6^ |
|  | Spain | 14.00 | 0.20 | 0.43 | 0.80 | 0.44 | 5.6x10^-6^ |
|  | **Overall** | **29.25** | **0.19** | **0.47** | **0.83** | **0.43** | **1.2x10^-6^** |
| ***C. tropicalis*** | Brazil | 8.66 | 0.13 | 0.46 | 0.69 | 0.31 | 6.6x10^-7^ |
|  | Denmark | 6.66 | 0.22 | 0.31 | 0.38 | 0.57 | 2.6x10^-6^ |
|  | Italy | 10.50 | 0.12 | 0.52 | 0.72 | 0.31 | 1.8x10^-7^ |
|  | Spain | 4.66 | 0.12 | 0.50 | 0.70 | 0.30 | 3.1x10^-6^ |
|  | **Overall** | **15.83** | **0.16** | **0.47** | **0.76** | **0.38** | **4.0x10^-8^** |

^a^Observed and expected heterozygosity ranged from 0 (no heterozygosity) to 1 (highest heterozygosity). ^b^Wright’s index indicates a deficiency of heterozygosity (positive values) or excess heterozygosity (negative values). ^c^The probability of identity indicates the likelihood of finding two identical genotypes after randomly selecting two isolates.
